# Clinical Society of Maryland

**Published:** 1887-08

**Authors:** 


### THE CLINICAL SOCIETY OF MARYLAND.

STATED MEETING, HELD May 5, 1887.

Dr. W. B. Platt exhibited a specimen of a tumor from the naso-pharynx, and also the patient from whom it was taken.

The tumor was removed from the naso-pharynx. It began to manifest itself about two years previously. There was much pain on swallowing. Nose was stopped, principally on the left side. Palate pushed downward and the uvula was toward the right side. Hemorrhage took place once during the progress of the growth. Patient was sent to Bayview Hospital and the operation was performed. A longitudinal incision was made through the palate and the galvano-cautery wire was tried in order to secure the tumor, but it failed. Then the coal steel wire was used, the tumor secured and removed with very little hemorrhage. The wound was stitched together and remained so for nine days. Patient recovered well and her speech was very much improved. He thought first of making a transverse incision, but the longitudinal one has the advantage of giving more room to work. The tumor was irregular in shape and quite firm. The question is, should he not have done a more radical operation? Hemorrhage is sometimes great in these cases. Dr. Sands, of New York, once removed a tumor with forceps and there was great hemorrhage from it.

Dr. Hiram Woods read a paper entitled

### REVIEW OF RECENT LITERATURE ON CHLOROFORM AND ETHER.

In the discussion which followed, Dr. G. J. Preston said he felt very much interested in the paper of Dr. Woods. He remembered a discussion in this Society a few years ago, when chloroform was decided to be the better agent. The physiological action of the two drugs is an important factor. With chloroform the effects are sudden. With ether we can see the danger coming. The fact that oculists have been so successful with chloroform is due to the stimulating effects which chloroform first produces and that the operations are short. He thinks that the cases should be selected for the anæsthetic. Where there is bronchitis and other affections of the air-passages chloroform should be given. For prolonged operations without these complications, ether is best.

Dr. J. G. Wiltshire said he was very much pleased to hear the paper. Chloroform depresses the heart and stimulants are indicated. If chloroform inhibits the action of the pneumogastric nerve, does not atropia stimulate the excitor motors?

Dr. Robert Johnson said such discussions should come up every year, because administrators of anæsthetics become so taken up with the one that they use that they become careless as regards their danger. He called attention to two cases. One was a case of hare-lip. He gave chloroform and the child died on the fourth day with capillary bronchitis. The other case was a man with Bright's disease, to whom he gave ether, and he made a good recovery. Atropia stimulates the respiratory centers; amyl nitrate is also a good stimulant.

Dr. J. T. Smith said the subject under discussion is an old one, but it is constantly recurring. The tide fluctuates in both directions. When men of great experience give such diverse opinions, both agents must be good. Ether is now more used than formerly; so more deaths have been reported. The safety of anæsthetics in ophthalmic practice is due to the fact that the operator is at the face of the patient and can observe more clearly any changes that might take place. He was glad that the subject was brought forward for discussion.

Dr. W. B. Platt said ether had won its way into England, where at one time prejudice was greatly against it. Chloroform is used more in Germany. Morphia was advocated by Professor Tieste, of Leipsic. It was thought by its use less anæsthesia would be required. There will always be some cranks against the use of one or the other of the anæsthetics, just as similar individuals oppose vaccination, for example. He thinks that he will soon settle down to the use of ether again.

Dr. G. H. Rohé said that he had had a good deal of experience with ether and never had failed to anæsthetize his patient. He had one patient to die on the thrid day after operation for vesico-vaginal fistula from pneumonia. What the cause of the pneumonia was he could not say. He now always has patients well protected by covering during ether administration. He never believed in Dr. Emmet's statement about the danger of ether in Bright's disease. He believes it is not good to give it in bronchitis. Ether increases the bronchial secretions and the accumulations may be so great in the lungs as not to be thrown off. He believes that Dr. Sayre's method of giving saturated chloroform vapor is a dangerous one. Death usually takes place upon the addition of more chloroform. Both ether and chloroform depress the heart, as sphygmographic tracings have shown. He has not given alcohol for ten years, but uses morphia and atrophia before the anæsthetic. It reduces the reflex irritability of the larynx to the ether vapor.

Dr. W. P. Chunn said he had given both anæsthetics and never saw a life lost. Five or six years ago chloroform was in more common use than ether. Chloroform is more powerful in its effects, and likewise more rapid, consequently more dangerous. Even if the chloroform is stopped before the muscles are relaxed, anæsthesia will increase for a time. Chloroform in certain cases is fairly safe. A great deal depends on how it is given. In regard to ether he had never seen an accident.

Dr. W. H. Norris thought that a great deal of the trouble which comes on from anæsthetics is due to the quality of the drug. He had given chloroform many times, and he had only seen one case where it acted badly. Chloroform was used during the late war by all army surgeons, and they agreed that it was the best anæsthetic. He had had little experience with the use of ether. He related a case of puerperal convulsions where he gave four ounces without effect. He asked the opinion of the members as to the use of the combination of chloroform and ether.

Dr. W. Winsey said as to which is the safer of the two drugs, those who use them will be guarded by the effects of either. He prefers ether. A patient under the influence of an anæsthetic is never free from danger, the administration of which should only be entrusted to competent hands.

Dr. C. W. Mitchell said he had given both agents for the past seven years, though chloroform has been in more general use. He began its use at the Presbyterian Eye and Ear Hospital. He finds now that ether is more generally given than chloroform. Anæsthetics should be given as other drugs are; ether is not comparatively safer nor comparatively more dangerous. Care should be practiced in the selection of cases. He had seen two deaths from the effects of each drug. The fact of concentration should be considered in ether as well as in chloroform. The bronchial secretions, increased by the influence of ether, should be allowed to escape by turning the head to one side and pulling the lips apart by placing the fingers in the patient's mouth. The snoring from ether is the result of the accumulation of mucus in the pharynx. Of the two deaths from chloroform, in the first the drug was givin by an inhaling apparatus, and only ⅔ of one teaspoonful was used; in the second case, a towel was used for the administration, and the chloroform was only poured on once; both patients died suddenly. The first case of death from ether the patient died on the ninth day from pneumonia. He thinks that chloroform would have been better. The second case was a patient with cavities in both lungs, upon whom laparotomy was performed. The fifth day afterwards acute pulmonary trouble came on, producing severe pulmonary hemorrhrge and finally death. Kidney trouble renders both drugs dangerous.

Dr. L. E. Neale was glad to observe the practical turn which Dr. Mitchell had given to the discussion in trying to select special anæsthetics for special cases and special conditions. Just here he thought it interesting and important to note that chloroform had thus far held the lead as an anæsthetic during parturition, and out of many hundred thousand times it has been given, not a single well-authenticated case of death is on record. This was possibly explained by the physiological hypertrophy of the heart preventing cerebral anæmia.

Dr. L. McLane Tiffany said the question is not between chloroform and ether, but when we have to produce anæsthesia, how can we best bring it about? It is wrong to think we have only two drugs to select from. The recognition of the disease, the choice of the remedy and its application, are the essential points to be considered. There is no such thing as best anæsthetic. Then it comes to the indications; contra-indications to the use of ether rests largely in the lungs. It has occurred to him to change the anæsthetic even while operating. Chloroform is better in such conditions. He related a case where he removed a tumor from a patient's neck. He began anæsthesia with ether; the secretions from the bronchial tubes began to pour out so fast that the ether was discontinued and chloroform used instead. The patient did well after the change. In the aged, say 70 years, he always gives chloroform, and also in the young. In all other cases, other things being equal, he gives ether. He then related a case where the patient had both bronchitis and heart trouble. He gave chloroform and the patient did well. He always precedes the anæsthetic with morphia and atropia.

Dr. J. E. Michael said it is fortunate that this subject does recur for discussion. The tide is very apt to turn with experience. He is not given to ether alone nor to chloroform, for experience shows that both are dangerous and must be carefully watched. In both cases of death from chloroform reported by Dr. Mitchell there was found disease of the kidneys. If other things are equal in a patient with kidney disease, he does not know which he would choose of the two anæsthetics; where there is much bronchial secretions ether is contra-indicated. Chloroform is best in tracheotomy. Select the anæsthetic according to the indications. The death from ether in the case related by Dr. Mitchell was caused by hemorrhage which it excited. These discussions will compel us to look at the matter from a clinical standpoint. Tendency seems decidedly towards reviewing the ground and to determine which is the better agent to use under certain circumstances.

Dr. R. Winslow said that he was struck while abroad at the faulty methods employed in Billroth's clinic in administering the A. C. E. mixture. It was frequently given in the upright position. One patient died from such methods of administration.

Dr. Hiram Woods, in conclusion, said: What has been said by the gentlemen who have spoken recalled a remark he heard Dr. Tiffany make in this Society about four years ago when this same subject was under discussion. He said: "No matter what anæsthetic you give, you put the patient in a position greatly resembling death, and it always needs care to avoid stepping over the line." The mainstay in the use of any anæsthetic is the recognition of danger. Drs. Preston and Smith have alluded to the pallor of the face as being more apt to be noticed when the operation is about that part of the body, and this, to an extent, explains why the oculist does not have many accidents. Another reason is that it is impossible for the operator to be at work on the eye while the anæsthetic is being given. Dr. Chisolm was the first he heard give this reason. When the operation begins, giving of the anæsthetic stops. If the patient awakens the anæsthetic has to be renewed; the operator stops, and as he wants to get back to his work as soon as possible, there is not much danger of the patient getting an overdose. Dr. Smith has also alluded to operations about the peritoneum as being specially dangerous. In the remarks made by Dr. Weir at the New York Academy, deaths during operations on the peritoneum are excluded by the speaker from deaths due to anæsthetics, because, I suppose, any injury to the peritoneum is so apt to be followed by shock. He thought Dr. Wiltshire must have misunderstood him, or he must have used the wrong work. Vulpian's experiments showed that anæsthetics increase, not decrease, the inhibition of the vagus ; hence atropia antagonized them. As the doctor says, part of the good effects of atropia undoubtedly comes from the direct stimulation of the cardiac ganglia. Dr. Johnson's case of capillary bronchitis following the use of chloroform in an operation for hare-lip and cleft-palate is open to the objection that the operation involved the respiratory track. Dr. Gerster thinks that all such operations should be excluded when we are speaking of the effects of anæsthetic agents on the lungs. The reason for the occurrence of lung troubles after operations was thought by Dr. Weir to be due to free use of antiseptics in surgical practice and the prolonged exposure of the patient. Dr. Platt says the use of morphia is not as common abroad as it formerly was. It has been found that large doses depress the heart and add to the danger. This is just what one would expect from large doses. The larger the dose the more transient is the stage of stimulation, and more profound the stage of depression. The dose should be small, not over $\frac{1}{8}$ gr. to $\frac{1}{4}$ gr. at most. Drs. Mitchell and Rohé each report fatal cases of pneumonia after the use of ether. Dr. Mitchell says the pneumonia is always catarrhal. One of the cases reported by Dr. Gerster was lobar pneumonia in the stage of engorgement. Neither of these gentlemen think that ether is contra-indicated in nephritis. He knows nothing of ether experimentally, but a reference to Dr. Gerster's paper in the *Medical Record* will show that many cases have occurred which show that ether is very dangerous in Bright's disease. He could not see how Dr. Rohé can find in Dr. Sayre's remarks teaching which advises the administration of *a saturated* chloroform atmosphere. Dr. Sayre gives a summary of the principles he advanced in 1876, which are substantially the same as those which he holds now. He is not acquainted with the article which Dr. Rohé says was published by Dr. Sayre some years ago, in which he recommends the administration of saturated vapor. His present paper, and the exrtact he gives from his teachings in 1876, are directly against such practice. He agreed with Drs. Rohé and Chunn that many of the chloroform accidents occur just after more chloroform is poured on the towel. He believed this so because the anæsthetizer often puts the towel with the fresh chloroform just where he had it before. Dr. Sayre's method of using an inhaler prevents this accident from occurring. As regards Dr. Pancoast's case, he did not wish to acquit chloroform of its share of the work. All that he claimed was that it is clearly shown that Dr. Pancoast gives chloroform for slight operations in a manner which many recognized authorities consider wrong. If the interview from the daily papers which he quoted is correct, the man died apparently from shock due to the performance of a slight but painful operation during partial anæsthesia. There was of course more danger under the chloroform than there would have been without any anæsthetic. There was also more danger than there would have been had the chloroform been pushed to full narcosis.

